# Kirke’s Hand-Book of Physiology

**Published:** 1889-05

**Authors:** 


**Kirke's Hand-Book of Physiology.** By W. MORRANT BAKER, F. R. C. S., Surgeon to St. Bartholomew's Hospital, late Lecturer on Physiology St. Bartholomew's Hospital, etc., etc., and VINCENT DORMER HARRIS, M. D., London, Fellow of the Royal College of Physicians; Demonstrator of Physiology at St. Bartholomew's Hospital, etc., etc. Twelfth edition. Rearranged, revised and rewritten, with five hundred illustrations. Cloth, 8vo, pp. xi., 784. New York: William Wood & Co., 1889.

This valuable text-book has now reached a twelfth edition, and therefore any extended notice would be superfluous. The book is too well and favorably known to require more than a few remarks concerning the changes and improvements over the other editions. It has been rearranged, thoroughly revised, and part of it entirely rewritten. The purpose has been to include the latest teachings regarding the important subject of physiology, and to make it a reliable guide for students. This purpose has been accomplished, and the work is fully abreast of the times in most particulars.

The authors announce the following changes in this edition: The sections on the Heart, the Blood, and the Muscular System have been greatly altered; while the chapters on the Nervous System, the Reproductive Organs, and on Development, have been completely revised and to a large extent rewritten. Some fifty new illustrations have been added. The work has been printed in two kinds of type, the smaller containing some details that may be omitted by junior students at a first reading. In this way its value as a text-book is increased.

The illustrations are very fine, and render material assistance. The temptation is great to review the book extensively, but this has been done so many times already, that, as has been suggested, it is unnecessary. We advise all students who wish to possess a text-book on Physiology that is at the same time comprehensive and succinct, to examine the merits of this one before purchasing.

The publishers' part has been well done, and they deserve a word of praise for making an attractive volume.

